# Study of an Energy-Harvesting Damper Based on Magnetic Interaction

**DOI:** 10.3390/s22207865

**Published:** 2022-10-16

**Authors:** Susana Aberturas, Antonio Hernando, José Luis Olazagoitia, Miguel Ángel García

**Affiliations:** 1 Industrial Engineering and Automotive Department, Nebrija University, Sta. Cruz de Marcenado, 27, 28015 Madrid, Spain; 2 Instituto de Magnetismo Aplicado (IMA), UXM, ADIF, 28230 Las Rozas, Spain; 3 Donostia International Physics Center, 20028 Donostia, Spain; 4 IMDEA Nanociencia, 28049 Madrid, Spain; 5 Instituto de Cerámica y Vidrio, Campus de Cantoblanco, CSIC, 28049 Madrid, Spain

**Keywords:** electromagnetic suspension, energy-harvesting shock absorber, permanent magnets, coils, electromotive force, experimental method, optimized design

## Abstract

The saving and re-use of energy has acquired great relevance in recent years, being of great importance in the automotive sector. In the literature, it is possible to find different proposals for energy-harvesting damper systems (EHSA)—the electromagnetic damper being a highly recurrent but still poorly defined proposal. This article specifically focuses on studying the concept and feasibility of an electromagnetic suspension system that is capable of recovering energy, using a damper formed by permanent magnets and a system of coils that collect the electromotive force generated by the variation of the magnetic field. To study the feasibility of the system, it is necessary to know the maximum energy that can be recovered through the winding system; however, the difficulties in obtaining the derivative of the magnetic flux and its derivative for each position make the analytical method very tedious. This paper presents an experimental method with which to maximize energy recovery by defining the optimum relative position between magnet and coil.

## 1. Introduction

At the present time, energy is essential to understand the concept of the welfare society as we know it. In recent decades, energy consumption has grown significantly and steadily, at the same time that the standard of living has grown and evolved [1].

Within the different existing types, the most-consumed energy source in the world is oil, key to the automotive industry, which, in addition to being a non-renewable energy, also emits a large amount of polluting gases into the atmosphere [2].

For these reasons and thanks to the development of new technologies, the electric vehicle seems to be a good candidate to lead the future of the automobile, a fact that is moving closer and closer and which can practically be considered the present.

However, the full implementation of electric vehicles seems to have some limitations, among which we can highlight its low autonomy compared to fossil fuel vehicles. To solve this problem, several options have been proposed; the most direct way to increase the autonomy of the electric vehicle is to increase the size of the battery, however, this means increasing the weight and size of the vehicle, which translates into a reduction in performance. Due to this drawback, this solution is not cost-effective, so other ways of increasing range have been investigated. A good option seems to be energy-recovery methods, a solution that not only reduces energy consumption and increases the vehicle’s range, but could also reduce the pollutant gases emitted.

In recent years, numerous researchers have focused on developing these systems, resulting, for example, in the well-known regenerative braking systems, which recover the energy generated during the vehicle’s braking action. There are different vehicle systems that dissipate energy, but it is advisable to look for those that dissipate the most energy and, therefore, those that will allow us to recover a greater amount of energy [3].

Among the options that have been used to “recycle” energy from a vehicle, solutions such as the following can be found: energy recovery brakes [4], exhaust pipes with thermoelectric generation [5], energy generation from internal combustion gases in the engine [6], energy generation with piezoelectric materials in the tires [7], energy recovery in the suspensions [8], etc.

Attention has been paid to magnetic fluid-based devices that can act as passive or active fluids assisting in the generation of different grades of damping power, reducing friction and assisting in heat dissipation, in order to avoid an increase in temperature from non-recovered energy. Significant work has also been carried out on magnetorheological dampers [9,10], and even with energy-harvesting ability [11]. Some studies can also be found on ferrofluids and their application on damping [12], their application to energy harvesting [13], and for their use in automotive applications [14].

In this article, we have chosen to study the suspension system; this system receives vibrations produced by the irregularities of the terrain and, thanks to the shock absorber, reduces the vibrations transmitted to the bodywork, transforming this kinetic energy into heat dissipated to the environment.

As is well known, shock absorbers have a fundamental function in the vehicle, which is to always keep it in contact with the road and, at the same time, to dampen the vibrations from the irregularities of the terrain which are transmitted to the occupants of the vehicle. Today’s shock absorbers in vehicles act passively. They are compact and relatively cheap elements, with the only drawback being that they receive mechanical energy from the vertical inertias of the vehicle and transform it into useless energy, dissipating it in the form of heat to the surrounding environment. This transformation is performed by forcing the viscous fluid inside the shock absorber to pass through narrow holes.

In recent times, consideration has been given to the possibility of trying to recover the mechanical energy that reaches the shock absorbers and, instead of transforming it into heat, restoring its usefulness. The so-called Energy-Harvesting Shock Absorbers (EHSA) perform this function, recovering the mechanical energy from the suspension and transforming it into electrical energy, renewing its availability to the vehicle’s auxiliary or power supply systems and increasing the vehicle’s overall efficiency.

In energy recovery in car shock absorbers, various ideas have been presented, either using piezoelectric materials (e.g., Xie et al. [15]) or using linear (Bogdan Sapinski et al. [16]) or rotational electromagnetic generators. In rotational electric generators, a system is needed to convert the translational motion of the suspension to a rotational motion. These translational–rotational mechanisms can be: pinion–rack (Zuo et al. [17]); ball screw (Amati et al. [18]); and/or hydraulic transmission (Xu et al. [19]) or hydraulic electromagnetic shock absorber (HESA, Galluzzi Renato et al. [20]).

One of the biggest challenges of energy recovery systems presented in the relevant literature is that there is no single protocol for comparing the performance of these systems. However, a methodology has recently been presented [21] that allows for such a study and has been extended [22] to compare the performance between different recovery technologies for shock absorbers.

Energy recovery through electromechanical systems is very promising because, in addition to allowing the recovery of part of the energy that would otherwise be dissipated, it allows the suspension to be converted into an active suspension, making it possible to change its operating characteristics electrically [23]. Damper-based energy-recovery systems have mostly been applied to cars, but it is also possible to find some design, development and implementation on motorbikes [24].

It is extremely relevant to provide an estimation (in percentage of the fuel consumption) of how much energy can be recovered with the magnetic system here proposed. This estimation can be performed after estimating the kinetic energy associated with the vertical oscillations of the vehicle, under the following reasonable assumptions. Let us consider a car of 1200 kg in weight running at a speed of 100 km/h, and for which the suspension undergoes an up/down oscillation of 4 cm average amplitude. Such oscillation is considered to take place each 25 m of the trajectory. The work carried out at each suspension motion is of approximately 350 J. Therefore, it reaches a value of 1.4 MJ in 100 km, which accounts for 392 W of average power recovery. This is an average and basic estimate that is in line with different studies that have analyzed the power recoverable in an energy-recovering suspension. Essentially, the power recovered depends strongly on the quality of the track on which the vehicle runs. Knowing that approximately one liter of gasoline has an energy of 36 MJ and that the efficiency of a combustion vehicle never exceeds 30%, the vehicle in our example would recover energy similar to 129 mL of gasoline, as the second principle of thermodynamics prevents this amount of energy from being fully conversed by the damper into electric energy. It is the design of the full magnetic damper that determines the optimum conditions for improving its efficiency. Even by considering the energy dissipated by the Joule effect in the coils system, the research effort oriented to elucidate the optimal design becomes justified.

The general objective of the research directly related to this article is to study the feasibility of a suspension system formed by permanent magnets, which, instead of dissipating energy in the form of heat, collects this kinetic energy and transforms it into electrical energy, which can be reused or stored. The design of this system is mainly composed of permanent magnets and coils [25]. The magnets must have a high energy product, such as NdFeB5, and must be placed in opposition, so that the repulsive force between them fulfills the function of a spring in the case of a conventional damping system. On the other hand, it is necessary to have one or more coils that, in the face of the flux variation produced by the movement of the magnet, collect this potential, thus, generating an electric current that will circulate through the coils.

The amount of energy collected by the coil will depend on the position of the coil with respect to the magnet; therefore, this factor is key in the design of our model. In this article, we study the field produced by a solenoid, the voltage collected by the secondary coil as a function of its position and in which positions the gradients are maximum, by means of experimental and theoretical measurements.

That is to say, the specific objective of this article is to determine a method to find the elements that maximize the energy collection and the optimal position at which the magnet and the energy-collecting coil should be placed. Since the analytical method to obtain the flux and its derivative for each position is very tedious, we focus especially on the experimental method.

## 2. Theoretical Approach

A permanent magnet fixed to the main damper of a vehicle can generate an electromotive force in a pick-up coil placed in its surrounding. This magnetic spring suspension gives rise to energy-recovery capabilities. The value of the electromotive force, *E*, is that given by Faraday’s law; *E* = −(*dϕ*/*dt*)) or the time derivative of the flux of the magnetic induction through the surface enclosed by the pick-up or secondary coil. When the secondary coil is inserted in a closed circuit with total resistance *R*, it becomes a current with intensity *I*_2_= (*E*/*R*) flowing along the circuit, and the absorbed power should be W = IE. This absorbed energy is provided by the kinetics energy of the suspension system and should give rise to dissipated heat and regenerative braking through the force exerted by the magnetic field produced by *I*_2_ on the magnet. Therefore, the maximum absorbed power takes place at the position of the secondary coil for which (*dϕ*/*dt*)) becomes maximum.

To optimize the design of the system, the position of the coil with respect to the magnet, oscillating along the *z*-axis, which maximizes (*dϕ*/*dt*)) and is compatible with the geometry and accessibility of the different positions in the real dispositive, should be determined.

The main objective of the magnetic damper design is to obtain the best position of the secondary coil, close to the *z*-axis, depending on the direction of the magnetic moment of the magnet. The difficulty of the task can be understood by considering the following issues.

An exact calculation of the flux requires computational procedures, as shown in the Appendix A, where a rigorous expression for the flux calculation is outlined. The main reasons for the indicated difficulty can be summarized as follows:

- Only in a few cases can the field produced by the magnet in the points forming the surface of the secondary coil be analytically expressed.
- The calculation of the flux would require knowing the analytical expression of the field for any point of the coil surface.
- During the magnet motion, the magnetic field in those exceptional positions admitting an analytical formulation loses this capability when changing position.
- Moreover, the oscillation of the magnet far from harmonic becomes stochastic in amplitude and frequency, making the calculation of the time derivative impossible.

**Figure 1:**
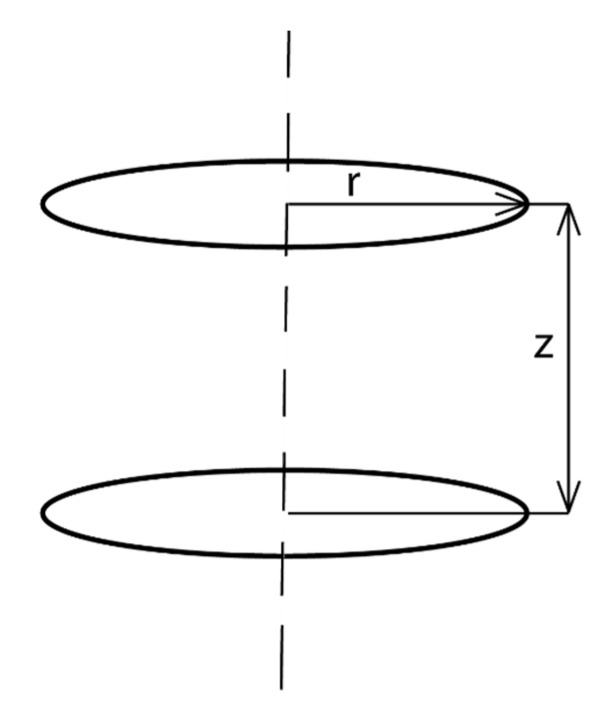
Diagram of z and r coordinates with respect to primary and secondary coils.

An example that illustrates these difficulties corresponds to the simplest case of two coaxial circular coils separated by a distance h. When a current of intensity I flows along one of them, the flux that crosses the other one is proportional to I, and the proportionality constant is known as mutual inductance M_1,2_. The calculation of M_1,2_ leads to a solution formed by elliptical functions that only can be calculated by numerical methods. This case coincides with the case of a magnetic suspension, in which the magnet magnetic moment lies along the z-axis and the normal direction of the secondary coil is also oriented along the same *z*-axis and is placed coaxially with the magnet (Figure 1). Notice that outside the magnet the magnetic field geometry is analogous to that produced by a coil located at the magnet position and with the same magnetic moment.

Hereinafter, we consider a circular primary coil coaxial with the *z*-axis and with intensity current *I*_1_ and area *s*_1_. The component of the magnetic field produced by the primary coil along the normal direction to the secondary coil can be expressed at any point of *s*_2_ as *B* = *μ*_0_*I*_1_*f* (*z*,*r*), where *f* (*z*,*r*) accounts for the dependence of the magnetic field with the distance between the coils and the relative orientation. Let us suppose that the primary coil (or the magnet) fixed to the suspension system oscillates around the point z* with amplitude *z*_0_ and frequency *ω*_2_. Therefore, *z* = z*+ *z*_0_ cos *ω*_2_*t*,

As *B* = *μ*_0_*I*_1_*f* (*z*,*r*) when *I*_1_ is constant, the change of the magnetic field component at any point of the surface of the secondary coil could be only due to changes in the relative position of the two coils, that is, changes of *z* or *r*. Consider that, because of the cylindrical symmetry, the magnetic field component contributing to the flux through the secondary coil does not change under rotation around the *z*-axis, i.e., it is independent of the *ϕ* angle. The flux crossing the secondary coil becomes

(1) $$\phi =n\mu_{0}I_{1}\iint \;f\left(z,r\right)ds_{2}=n\mu_{0}I_{1}g\left(z,r\right)$$

Thereby, the amplitude of the sinusoidal electromotive force induced in the secondary coil is

(2) $$E=\frac{-d\phi }{dt}=n\mu_{0}I_{1}\omega_{2}z_{0}\frac{dg\left(z,r\right)}{dz}$$

The position of the secondary coil that maximizes E is that for which $\frac{dg\left(z,r\right)}{dz}$ reaches a maximum. However, the calculation of *g* (*z*,*r*) first requires the calculation of *f* (*z*,*r*), a function that is analytical in very few cases, and second, the integration of *f* (*z*,*r*) over *s*2.

Nevertheless, we propose here an experimental method that overcomes these difficulties. An ac current *I* = $I_{0}cos\omega_{1}t$ flows along the primary coil that can be located at different points with respect to the secondary coil. Now, suppose that we fix the relative position of the two coils, *B* = *μ*_0_*If* (*z*,*r*), but *z* and *r* are fixed; then, the flux through the secondary is given by:

(3) $$\phi =n\mu_{0}I_{0}cos\omega_{1}t\iint \;f\left(z,r\right)ds_{2}=n\mu_{0}I_{0}cos\left(\omega_{1}t\right)g\left(z,r\right)$$

The electromotive force induced in the secondary coil is now:

(4) $$E=\frac{-d\phi }{dt}=n\mu_{0}I_{0}\omega_{1}g\left(z,r\right)\phi \;E=n\mu_{0}I_{0}cos\omega_{1}t\iint \;f\left(z,r\right)ds_{2}=n\mu_{0}I_{0}cos\left(\omega_{1}t\right)g\left(z,r\right)$$

*E* can be easily measured by using a frequency $\omega_{1}$ that is high enough to obtain a well-defined signal at different relative positions of the two coils. As *E* is proportional to g(z,r), the plot of the experimental *E* with the change of the component *z* of the relative position allows us to determine *dg* (*z*,*r*)/*dz*, as well as the positions for which this function reaches a maximum.

From these values of the maximum, it is possible to infer the power absorbed by the secondary coil when it is located close to an oscillating primary coil or magnet, which moves following the stochastic motion of the suspension system

## 3. Experimental Method

Although the results obtained with a magnet instead of a primary coil are not similar, the spatial distribution of the field is similar. This means that the optimum position will be the same in both cases.

The relevant field component is that which is parallel to the pick-up coil axis, which should change in time in order to induce an electromotive force. The change can be due to a change in the relative position of the pick-up coil with respect to the field source (oscillating source), or a change in the intensity current when the source is a fixed primary coil.

**Figure 2:**
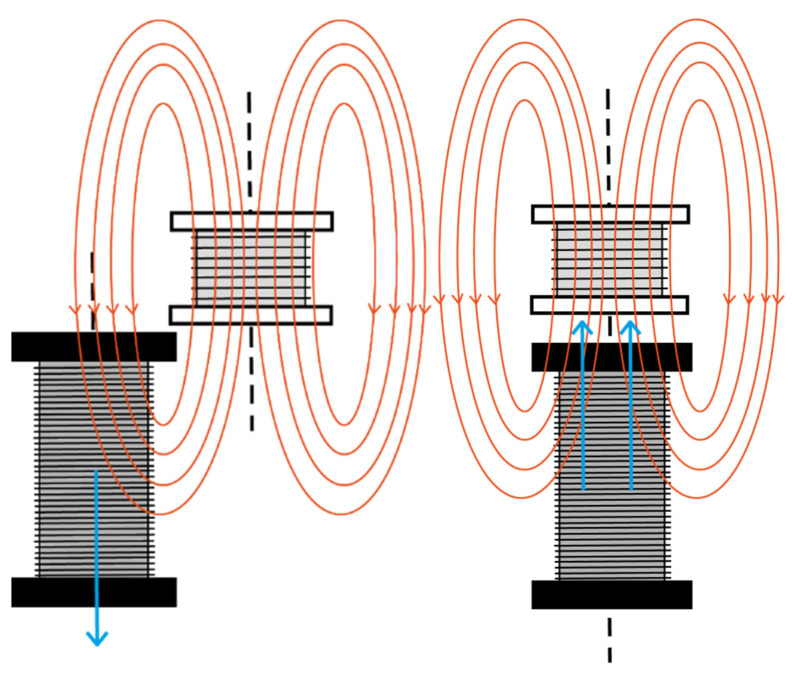
Induced voltage placing the secondary coil with its axial axis vertical.

We have considered the magnetic moment to be oriented along the oscillation direction. In this case, the pick-up coil axis can be oriented between two limiting cases, parallel (Figure 2) or perpendicular to the magnetic moment. Both cases are analyzed experimentally. However, a similar procedure can be used when the magnetic moment is perpendicular to the oscillating direction, provided that the component of the field parallel to the pick-up axis oscillates in time.

Measurements were performed by varying the z component; in addition, depending also on the r component, the secondary will collect flux depending on the number of field lines crossing the pick-up coil.

Precise tests are carried out with the help of a Lock-in amplifier, and the positions in which the greatest flux variation is obtained are checked. For this purpose, a primary coil of 150 turns is used, through which an alternating current of 5 volts peak-peak at a frequency of 1 kHz is passed. Further, a secondary coil is used, which is connected to the Lock-in to obtain the voltage induced in this coil as a function of the position with respect to the primary coil.

First, we place the secondary coil with its axis vertical, so that the voltage can be collected as shown in the following Figure 2.

**Figure 3:**
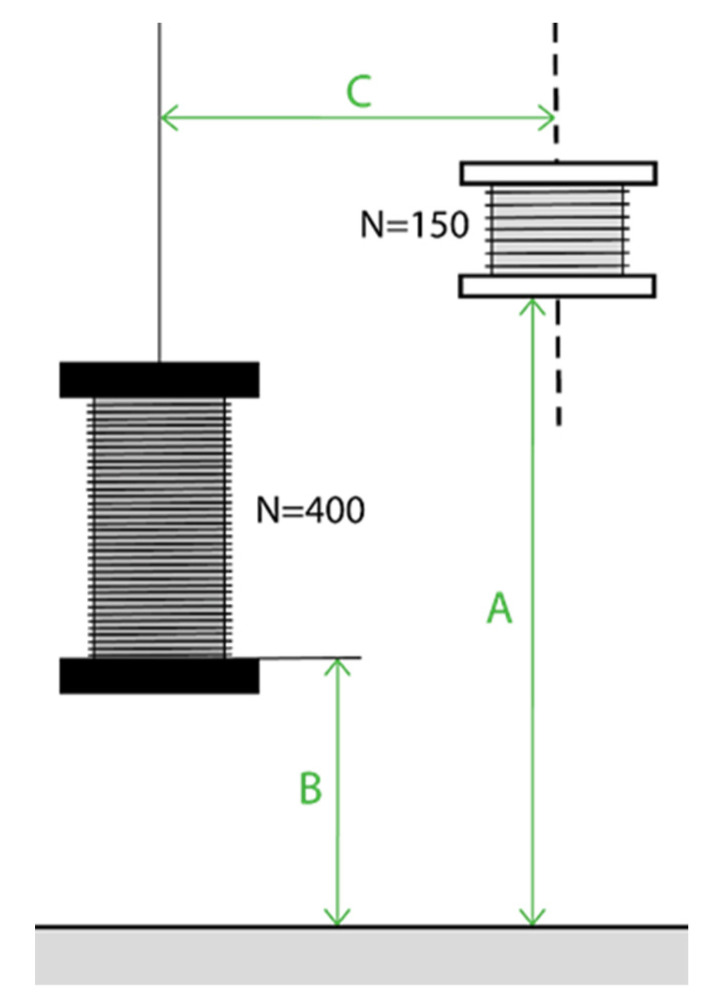
Diagram of the position parameters A, B and C to be varied during Test 4.

Returning to the method and equations explained in the theoretical approach the equations used in case 1 are summarized on Table 1:

Different trajectories are tested by varying the values of A and C shown in Figure 3.

The voltage measurements taken below represent the FEM term, which is measured in mV:

After the tests are carried out, the variations obtained in the case of placing the secondary coil in a horizontal position are measured, since the way in which the coil collects the flux varies.

As we can see in the image on the right below, this arrangement of the coils does not generate flux variation, since the direction of the reactions is the same, but in the opposite direction.

Next, by taking measurements experimentally, we study how the voltage induced in the secondary coil varies according to the different values of the variables A and C.

Returning to the method and equations explained in the theoretical approach the equations used in case 2 are summarized on Table 2:

After taking the previous measurements and plotting them in order to draw conclusions about which is the best option to design our prototype, we decided to take further measurements.

**Figure 4:**
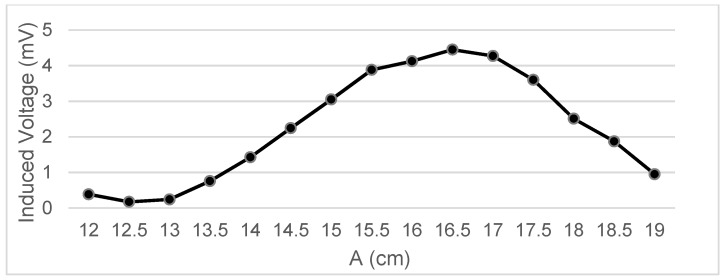
Data representation for B = 16 cm, C = 5.5 cm; Test 4.1.

**Figure 5:**
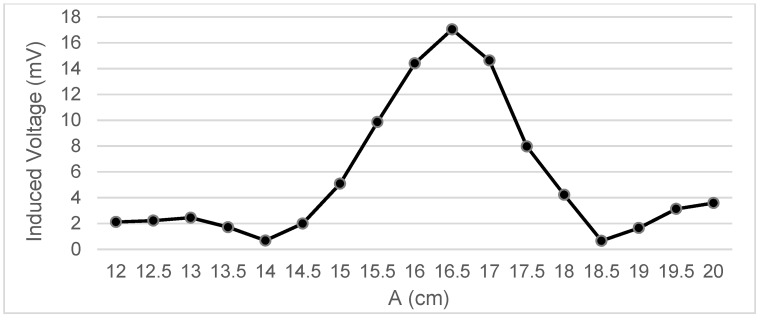
Data representation for B = 16 cm, C = 4 cm; Test 4.2.

**Figure 6:**
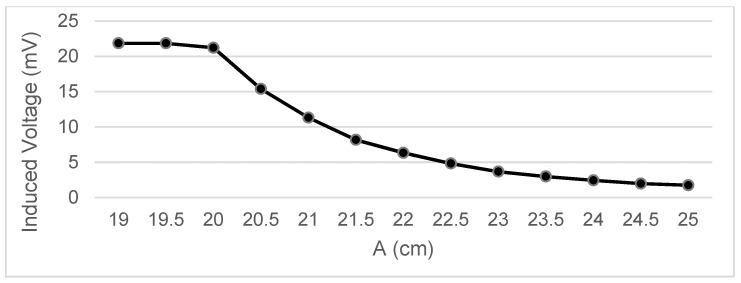
Data representation for B = 16 cm, C= 0 cm; Test 4.3.

**Figure 7:**
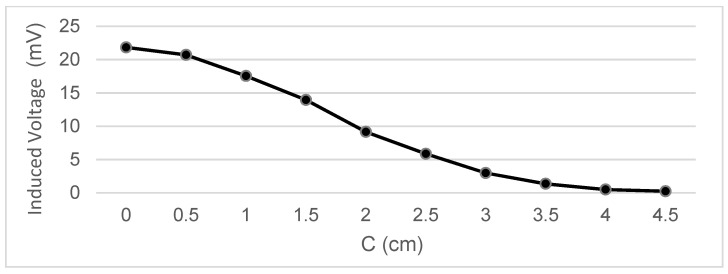
Data representation for B = 16 cm, A = 20 cm; Test 4.4.

**Figure 8:**
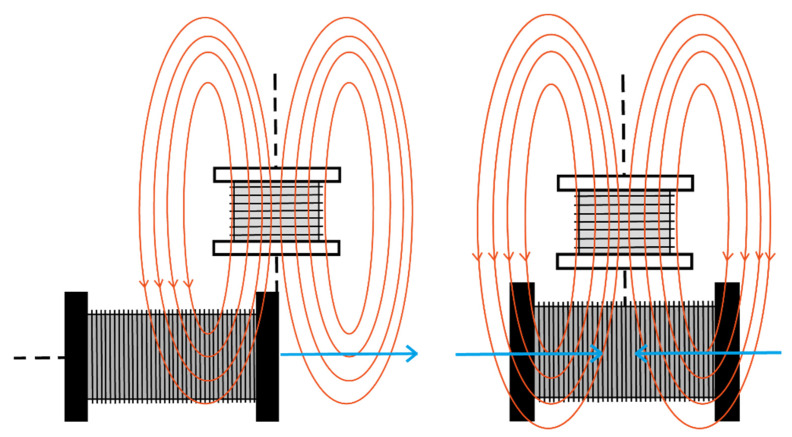
Induced voltage placing the secondary coil with its axial axis horizontal.

**Figure 9:**
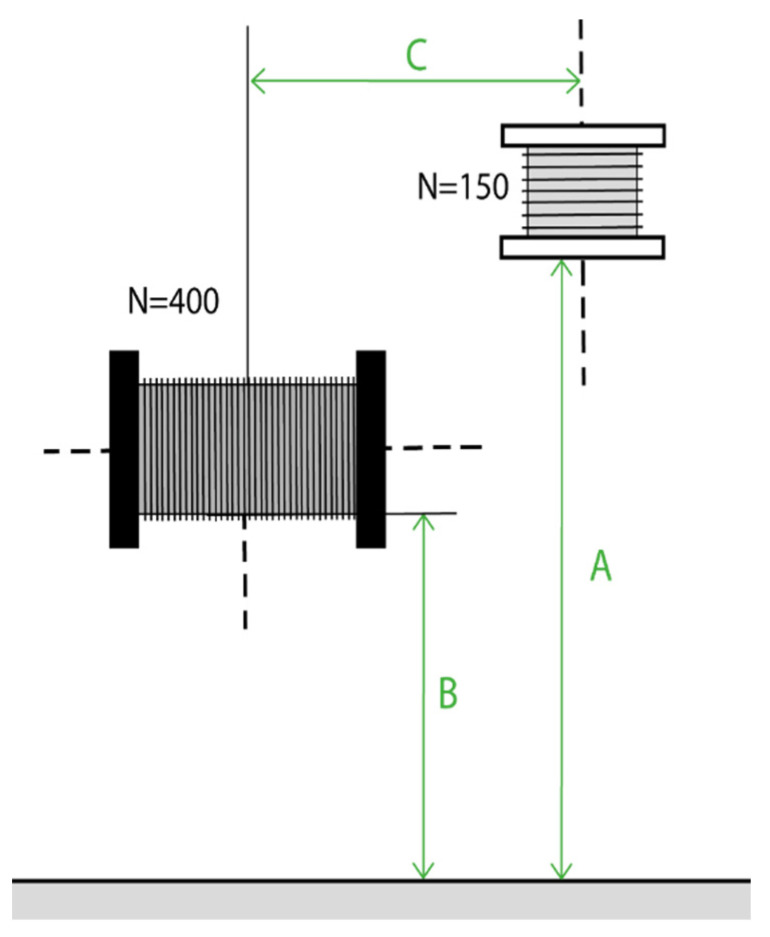
Diagram of the parameters to be varied during Test 4, horizontal arrangement.

**Figure 10:**
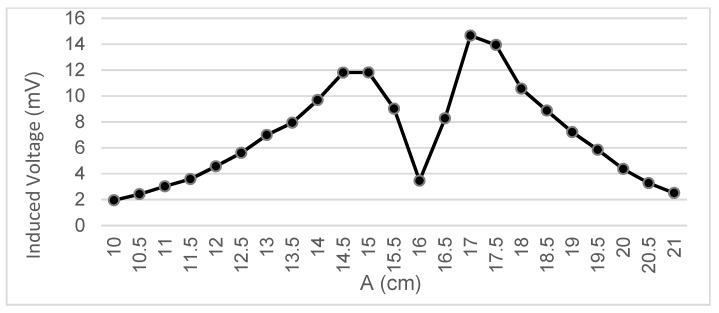
Data representation for B = 15 m, C = 2.35 cm; Test 4.5.

**Figure 11:**
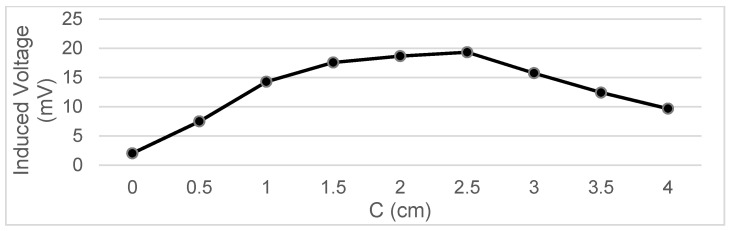
Data representation for B = 15 cm, A = 18 cm; Test 4.6.

**Figure 12:**
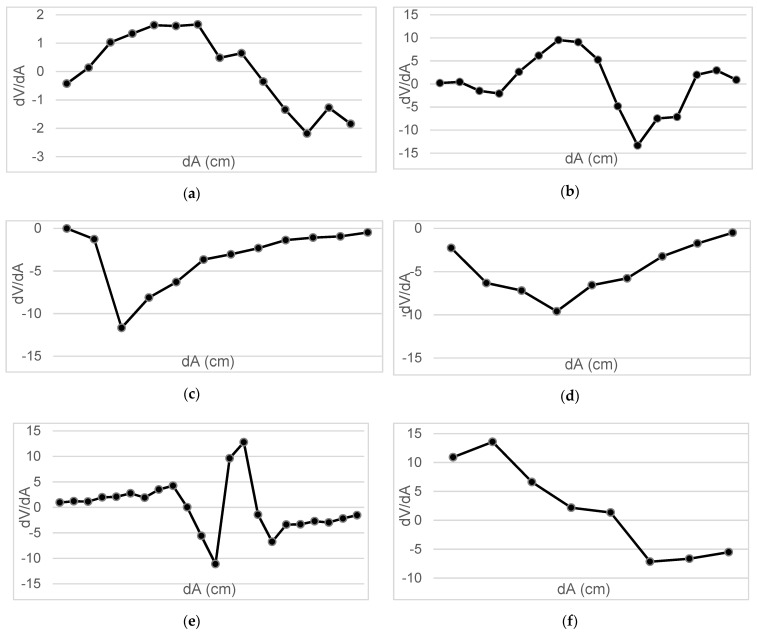
(**a**) Gradients Test 4.1; (**b**) Gradients Test 4.2; (**c**) Gradients Test 4.3; (**d**) Gradients Test 4.4; (**e**) Gradients Test 4.5; (**f**) Gradients Test 4.6.

Measurements were taken of the voltage induced in the secondary coil in positions that were 5 mm apart. The calculated induced voltage is shown in Figure 4, Figure 5, Figure 6 and Figure 7 for parallel coil axes. For perpendicular coil axes as shown in Figure 8 and Figure 9, the induced voltage is shown in Figure 10 and Figure 11. If the derivative of the functions is performed, it is possible to identify where the value of the derivative is higher, and it is in that position where the maximum flux variation is obtained. Therefore, the calculated gradients of the fields in Figure 12 change according to the variation of the induced voltage with the coil position. The gradient exhibits a maximum at the optimum place to place the coil for obtaining the maximum flux variation for a given movement of the magnet.

The objective is to observe the configuration that provides us with the maximum derivative, and within it, to determine in which section the highest variation of flow occurs.

Table 3 shows the maximum gradient of each test to check which is the point where the value *dg* (*z,r*)/*dz* is maximum:

The maximum gradient is the one corresponding to Test 4.6. By retaking the data collected in the tables of that test, it is observed (Table 4) that this maximum gradient corresponds to the position A = 18 cm, B = 15 cm, and a variation from C1 = 0.5 cm to C2 = 1 cm:

In the magnet–coil system integrated in the suspension of a vehicle, the magnet will travel a distance greater than the 0.5 cm at which this maximum variation occurs. For the future design of the system, this configuration could be repeated by placing several coils connected in series; in this way, this maximum variation will be collected during the whole magnet travel, thanks to several coils whose induced voltage will be summed up. In this case it is Test 2, in section A1= 16.5, A2 = 18.5, with a variation of 16.378 mV.

As the magnet in our system will travel a longer distance than the 2 cm where this maximum variation occurs, we can repeat this configuration by placing several coils connected in a series. In this way, this maximum variation will be collected during the whole travel of the magnet, thanks to several coils whose induced voltage will be added together.

**Figure 13:**
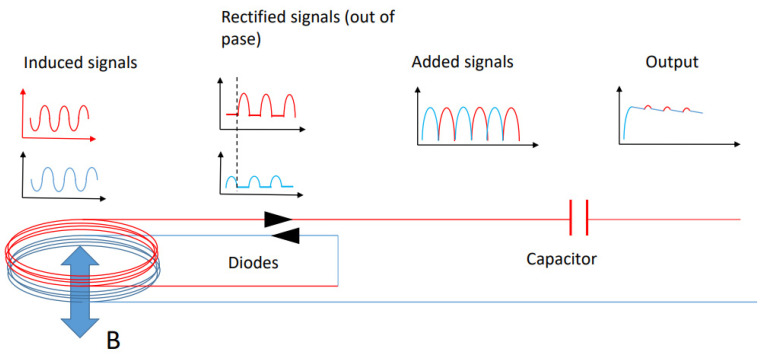
Damping rectifier diagram.

Finally, it should be added that the AC voltage provided by the system can be used to directly provide a DC current. A simple configuration for this purpose is to place together two coils and rectify their output with diodes in the opposite sense, so that rectified signals are de-phased. Then, the signals are added by appropriated connections and the output signal is passed through a capacitor with a time constant that is large enough to provide an almost-DC signal (Figure 13).

## 4. Conclusions

The experimental method described and applied in Section 3 and based on the theoretical approach depicted in Section 2 allows us to overcome the intrinsic difficulty enclosed in the numerical calculation of integrals (A3) and (A4), which yields the flux crossing the secondary coil and the induced electromotive force, respectively. Note that to choose the optimum position of the secondary coil for a subsequent damper construction, it is also necessary, once the flux has been calculated, to elucidate that position corresponding to the maximum flux gradient. The method suggested here also provides a rapid detection of the maximum gradient by studying the change of the induced voltage through displacing the secondary coil small distances around its initial position. In fact, due to the only influence of the relative positions of the two coils in the value of the mutual inductance, the experiments can be carried out by using the fixed coil as a primary or secondary one.

## Figures and Tables

**Table 1:** Summary table of equations, case 1.

CASE 1. AC COIL-COIL SYSTEM		
$B=\mu_{0}I\left(t\right)f\left(z,r\right)$	$\phi =N\mu_{0}I_{0}cos\left(\omega_{1}t\right)g\left(z,r\right)$	$FEM=N\mu_{0}I_{0}\omega_{1}sin\omega_{1}tg\left(z,r\right)$

**Table 2:** Summary table of equations, case 2.

CASE 2. DC COIL-COIL SYSTEM		
$B=\mu_{0}If\left(z\left(t\right),r\right)$	$\phi =N\mu_{0}Ig\left(z,r\right)$	$FEM=N\mu_{0}I\omega_{2}z_{0}sin\omega_{2}t\frac{dg\left(z,r\right)}{dz}$

**Table 3:** Maximum gradient of each test in absolute value, Test 4.

MAXIMUM GRADIENT OF EACH TEST					
TEST 4.1	TEST 4.2	TEST 4.3	TEST 4.4	TEST 4.5	TEST 4.6
1844	13,326	11,678	9602	12,774	13,566

**Table 4:** Comparison of optimal data, Test 4.6.

GRADIENTS TEST 6			
dV (mV)	dA (cm)	dV/dA	
5458	0.5	10,916	
6783	0.5	13,566	
TEST 4.6. B = 15 cm; A = 18 cm			
C (cm)	CHANNEL ONE (mV)	CHANNEL TWO (°)	REFERENCE (kHz)
0	2.03	−101.08	1
0.5	7488	83.34	1
1	14,271	82.88	1

## Appendix A

It is very common to study the magnetic field using the law of Biot and Savart, but this equation allows us to find the field on the axis of a loop (when the r component is equal to zero).

However, thanks to the equation of the vector potential or magnetic potential vector, we can know the magnetic field at any point in space, so this would be the most appropriate option for our case study.

The only component of the vector potential *A_φ_* created by a circular coil with radius *r*_0_ carrying a current of intensity *I*_0_, which at any point is located with respect to the coil at *r* and *z* in cylindrical coordinates, is given by

(A1) $$A_{\varphi }=\frac{{µ}_{0}I_{0}r_{0}}{2}\int_{0}^{\infty }e^{-k\left|z\right|}J_{1}\left(kr\right)J_{1}\left(kr_{0}\right)dk$$

As the magnetic field is the curl of *A*

(A2) B=(∇xA)

Then, Stokes’ theorem indicates that the magnetic flux crossing a circular secondary coil can be expressed as the integral along the secondary coil of *A_φ_*

(A3) $$\phi =\int \;A_{\varphi }\left(r’\right)dr{’}_{\varphi }=\int^{\;}\frac{{µ}_{0}I_{0}r_{0}}{2}\int_{0}^{\infty }e^{-k\left|z\right|}J_{1}\left(kr\right)J_{1}\left(kr_{0}\right)dkdr\prime_{\varphi }$$

where *r*′, is the position vector of the secondary coil points with respect to the center of the primary coil, which is considered as the origin of cylindrical coordinates. Note that the cylindrical coordinates of *dr*′ must be obtained through the equation of the curve that is defined by the secondary coil with respect to the origin. This integral (A3) must be generally obtained numerically.

In the case of a harvesting damper, the flux variation is originated by a change in the primary coil (or magnet) position along the *z*-axis. Therefore, the emf induced in the secondary coil, given by Equation (2), can be written as

(A4) $$E=\frac{{µ}_{0}I_{0}r_{0}}{2}\int^{\;}\int_{0}^{\infty }-ke^{-k\left|z\right|}J_{1}\left(kr\right)J_{1}\left(kr_{0}\right)\left(\frac{dz}{dt}\right)dkdr{´}_{\varphi }$$

When the primary coil motion is that of a harmonic oscillator, *z* = *z** + *z*_0_ *cos* *ω*_2_*t*, a comparison with relation (2) yields

(A5) $$\frac{dg\left(z,r\right)}{dz}=\frac{r_{0}}{2}\int^{\;}\int_{0}^{\infty }-ke^{-k\left|z\right|}J_{1}\left(kr\right)J_{1}\left(kr_{0}\right)dkdr{´}_{\varphi }$$
